# Effect of Plant Topping on Seasonal Development, Physiological Changes, and Grain Yield of Soybean

**DOI:** 10.3390/plants14132068

**Published:** 2025-07-06

**Authors:** Sora Lee, Chaelin Jo, Miri Choi, Jihyeon Lee, Nayoung Choi, Chaein Na

**Affiliations:** 1 Division of Applied Life Science, Gyeongsang National University, Jinju 52828, Republic of Korea; dlthfk010111@gnu.ac.kr (S.L.); jcl5220@gnu.ac.kr (C.J.); chlalfl0321@gnu.ac.kr (M.C.); 2 Allium Vegetable Research Center, National Institute of Horticultural and Herbal Science, Rural Development Administration, Muan 58545, Republic of Korea; dltla1264@korea.kr; 3 Future Agriculture Center, Kyung Nong Corporation, Gimje 54338, Republic of Korea; nychoi@dongoh.co.kr

**Keywords:** plant topping, pinching, soybean, chlorophyll fluorescence

## Abstract

Soybean (*Glycine max* L.) is vulnerable to environmental stresses, such as heavy rainfall and high winds, which promote lodging and reduce plant performance during the monsoon season. To mitigate these issues, we evaluated the effects of plant topping, a practice involving the removal of apical buds, on plant architecture, physiological traits, and grain yield in four soybean cultivars over two growing seasons (2021–2022). Plant topping was performed at the V6-7 stage by cutting 30–35 cm above the ground. Plant topping reduced plant height by up to 23.5% and decreased leaf area index (by 8.0–16.4%), potentially improving light penetration into the lower canopy. Although chlorophyll concentration declined temporarily (297.8 vs. 272.8 mg m^−2^ for non-topping vs. topping, respectively), NDVI remained stable, indicating delayed senescence. Chlorophyll fluorescence parameters revealed cultivar-specific stress responses, particularly in Taegwang, which showed elevated ABS/RC, TR_0_/RC, and DI_0_/CS values under plant topping. Grain yield was generally unaffected, except in Jinpung, which increased by 34% under plant topping in 2021 (2701 kg ha^−1^ vs. 3621 kg ha^−1^ for non-topping vs. topping). In conclusion, plant topping may help improve canopy structure and light distribution without compromising yield, potentially reducing lodging risk and offering a cultivar-specific management strategy.

## 1. Introduction

Soybean (*Glycine max* L.) is an annual field crop and a vital source of plant-based protein and oil [1]. It is widely utilized as both a staple food and animal feed [2]. Recently, soybean production has shown a declining trend in many regions due to the impacts of climate change [3]. Rising temperatures and variable rainfall are major factors directly affecting the growth and yield of soybean [4,5]. Elevated temperatures accelerate the flowering and maturity of soybean, thereby shortening the overall growing season [6], while extreme heat events impair pollination and pod set, leading to significant yield losses [7,8]. These environmental stresses have reduced yields in major soybean-producing regions, posing a threat to the stability of global soybean supply [9]. Particularly in South Korea, the domestic production rate of soybean has also decreased steadily, from 7.9% in 2011 to 5.9% in 2021 [10], while South Korea has become one of the major soybean-importing countries in the world [11]. To achieve sustainable domestic soybean production, improvements in current cultivation practices and the selection of ideal cultivars are required.

Plant topping, also referred to as terminal bud removal, pinching, or decapitation, is an agronomic practice involving the removal of the apical meristem at the tip of the stem or branches. This technique has gained attention as a promising approach to modify aboveground plant architecture to improve growth and yield without genetic modification [12]. Plant topping in soybean suppresses the growth of the main stem while promoting the development of lateral branches, contributing to the formation of a canopy structure that is favorable for photosynthesis [13]. After plant topping, new leaves develop from axillary buds, which can lead to an increase in the leaf area index (LAI) and delayed senescence, thereby maintaining leaf greenness [14,15]. This helps mitigate shading of the middle and lower leaves, which otherwise accelerates premature senescence and impairs nutrient translocation to reproductive organs [16]. As a result, the upper canopy exhibits more uniform leaf distribution, improving photosynthetic efficiency and ultimately enhancing crop growth [15].

The monsoon climate significantly influences soybean cultivation in Korea in early to mid-summer, which often results in heavy rainfall during the mid-growth stage. This excessive precipitation can lead to excessive stem elongation, thereby increasing susceptibility to lodging due to an unstable plant architecture [17]. In particular, during the late growth stages, the risk of lodging is further exacerbated by typhoons and other extreme weather events, which negatively impact yield and quality, ultimately reducing overall productivity [18,19]. High temperature and humidity conditions promote excessive internode elongation, thereby increasing the likelihood of lodging occurrence [20,21]. Plant architecture regulation through plant topping has gained attention to mitigate lodging. Plant topping primarily aims to improve plant architecture by suppressing apical dominance, thereby reducing the soybean plant height, preventing excessive elongation, and transitioning the plant into a structurally more stable form. These changes are advantageous in reducing the occurrence of lodging [22]. In the situation of cotton cultivation, plant topping has been shown to enhance the structural stability of the plant, which was associated with optimized resource allocation and improved lodging resistance [23].

Some researchers have explored plant topping as a potential method for yield improvement in leguminous crops [14,24]. By removing the apical bud, plant topping facilitates the development of lateral shoots, which can influence soybean growth and yield parameters [25]. These physiological responses are induced by the suppression of apical dominance, leading to a reduction in auxin concentration and a relative increase in cytokinin levels. Such hormonal shifts promote morphological and physiological changes, thereby improving the overall efficiency of resource utilization within the plant [23,26].

Plant topping of soybean was shown to increase the number of flowers and pods per node, ultimately improving grain yield [27]. However, the effects of plant topping are not always consistent. One study reported that plant topping extended the vegetative and reproductive growth phases but had no significant impact on grain yield [25]. It has also been suggested that the effectiveness of plant topping could be limited by certain factors, such as cultivar characteristics and planting density [28]. This highlights the importance of investigating how cultivar-specific traits affect plant responses to plant topping. Since cultivars can exhibit distinct growth patterns, resource allocation strategies, and yield potentials, a thorough understanding of these differences is crucial for optimizing agronomic practices and tailoring management strategies to improve productivity and sustainability [29]. However, a holistic understanding of plant aboveground performance after plant topping has not been fully examined for soybean plants.

The improvement in photosynthetic efficiency can be quantitatively evaluated using chlorophyll fluorescence, a useful tool for analyzing the electron transport status during photosynthesis and the plant’s response to light [30]. To analyze the effects of plant topping on different soybean cultivars, we employed the OJIP test, a non-destructive analysis that measures chlorophyll fluorescence responses by exposing dark-adapted leaves to light. This approach enables the evaluation of structural and functional changes in photosystem II (PSII) under stress conditions [31,32,33].

Thus, the current study investigated the effects of plant topping on various soybean cultivars, focusing on the aboveground growth and grain yield. The objectives of this study were as follows: (1) to elucidate the effects of plant topping treatments on the growth and physiological changes in soybeans, and (2) to identify the cultivar best-suited for plant topping treatments. By utilizing this approach, the study aims to provide insights into the physiological mechanisms underlying the effects of plant topping on soybean growth and yield, ultimately identifying cultivars best-suited for this agronomic practice.

## 2. Results

### 2.1. Weather Conditions

The monthly mean temperature, total rainfall, growing degree days (GDDs), total solar radiation, and duration of sunshine were measured during the soybean growing seasons from June to October in 2021 and 2022 (Table 1). The highest monthly mean temperatures were recorded in July, with 26.2 °C in 2021 and 26.5 °C in 2022. Total rainfall in 2021 was 948 mm, which was 38% higher than the 685 mm recorded in 2022. In both years, July had the highest monthly rainfall, with 359 mm in 2021 and 218 mm in 2022. The total GDDs were slightly higher in 2021 (1951 °C) than in 2022 (1896 °C), mainly due to elevated GDDs in October. Total solar radiation was 2520 MJ m^−2^ in 2021 and 2527 MJ m^−2^ in 2022. Similarly, the duration of sunshine was comparable, with 1009 h in 2021 and 1011 h in 2022.

### 2.2. Plant Height and Stem Diameter

The cultivar and plant topping effects on soybean plant height were compared in three developmental stages for two years (Table 2). At the V6 stage (before plant topping), Daewon had the highest plant height, while Daechan had the lowest for two years. After plant topping, the overall plant height was greater in 2022 than in 2021 across cultivars. There was a cultivar × plant topping interaction at the R1 stage for both years (*p* = 0.0380 in 2021; *p* = 0.0019 in 2022). At this stage, plant topping-treated plots had up to 20% lower plant height to the non-plant topping-treated plots in 2021, and 23.5% lower plant height in 2022 across cultivars. Daewon exhibited excessive elongation in non-plant topping-treated plots (control), but its plant height in plant topping-treated plots showed no significant difference from other cultivars in both years, except for Daechan in 2022. At later stages (R3 in 2021, R2 in 2022), two significant main effects for plant height were observed. Plant topping reduced plant height by 16.1% at the R3 stage in 2021 (75.0 cm vs. 62.9 cm) and by 14.6% at the R2 stage in 2022 (87.7 cm vs. 74.9 cm). The difference between plant topping-treated and non-plant topping-treated decreased at later growth stages compared to the R1 stage. There were some cultivar response differences in plant height. Daechan consistently had the lowest plant height among cultivars, while Daewon had the highest plant height in both years. Taegwang showed contrasting trends, exhibiting a relatively shorter plant height compared to other cultivars at R3 in 2021, but a relatively taller height at R2 in 2022.

The cultivar and plant topping effects on stem diameter were compared at three developmental stages for two years (Table 3). In 2021, no significant differences were observed. In 2022, there was a cultivar × plant topping interaction at the R1 stage (*p* = 0.0086). Except for Taegwang, three cultivars showed a lower stem diameter in the plant topping-treated plots compared to the non-plant topping plots. Taegwang exhibited the largest stem diameter under plant topping, while Daechan consistently had the smallest diameter among cultivars. Additionally, in 2022, significant differences in stem diameter among cultivars were observed at the V6 (before topping), R1, and R2 stages (*p* = 0.0058 for V6; *p* = 0.0001 for R1; *p* < 0.0001 for R2). Daewon and Taegwang exhibited the largest stem diameters for all stages.

### 2.3. LAI and NDVI

The effects of cultivar and plant topping on LAI were evaluated for two years (Table 4). Except for the R3 stage in 2022, plant topping significantly affected LAI at all growth stages for the two years. Notably, LAI was 15.1% lower at R2 (2021) stage and 10.7% lower at R1 (2022) in plant topping-treated plots compared to non-plant topping-treated plots (*p* = 0.0018; *p* = 0.0057, respectively), and this reduction continued into late stages—13.8% at R6 (2021) and 16.4% at R5 (2022) (*p* < 0.0001, *p* = 0.0002, respectively). Differences among cultivars were also evident. In 2021, Daechan and Jinpung had higher LAI values than Daewon and Taegwang at R4 (*p* = 0.0067), while Taegwang showed the lowest LAI at R6 (*p* = 0.0007). In 2022, cultivar differences reversed at R3, with Taegwang the highest and Daechan the lowest (*p* < 0.0001); Jinpung had the highest LAI at R5 (*p* = 0.0013). These findings indicate that the plant topping consistently reduced LAI across growth stages, and the magnitude of this reduction varied depending on the cultivar.

The NDVI was measured at three stages in 2021 and 2022 (Table 5). In both years, no significant differences in NDVI were observed among cultivars before plant topping. However, significant differences in NDVI were found among cultivars at the R2 stage in 2021 (*p* = 0.0340) and the R1 stage in 2022 (*p* < 0.0001). At the R2 stage in 2021, Daewon exhibited the highest NDVI, while Taegwang had the lowest. Conversely, at the R1 stage in 2022, Daechan recorded the lowest NDVI. At the R4 stage in 2021, NDVI was higher in plant topping-treated than in the non-plant topping-treated (0.893 vs. 0.888; *p* = 0.0028). Jinpung exhibited the highest NDVI at both R4 in 2021 and R3 in 2022, while Daechan consistently showed the lowest values (*p* < 0.0001). These results indicate that NDVI was influenced by both plant topping and cultivar.

### 2.4. Chlorophyll Concentration and Chlorophyll Fluorescence Parameters

Chlorophyll concentration was estimated at three different stages in 2021 and 2022 (Table 6). At the R1 stage in 2021, significant effects were observed for the cultivar and plant topping treatment (*p* = 0.0144; *p* = 0.0001, respectively). The chlorophyll concentration of plant topping-treated plots was lower than that of the non-plant topping-treated plots (272.8 vs. 297.8 mg m^−2^). Among cultivars, Daewon consistently maintained higher chlorophyll concentrations at the R1 and R3 stages in 2021, while Daechan tended to show lower levels. No significant differences were observed at the other stages.

To investigate the photochemical responses to plant topping treatment, various chlorophyll fluorescence parameters were analyzed at the R1 stage in 2021 and 2022 (Table 7). In 2021, F_v_/F_m_ and PI_ABS_ were not significantly affected. However, plant topping treatment × cultivar interaction was observed for ABS/RC, TR_0_/RC, and DI_0_/CS (*p* = 0.0089; *p* = 0.0047; *p* = 0.0243, respectively). In plant topping-treated plots, Taegwang exhibited the highest values for all three parameters, and notably, its values were also higher than those in the non-plant topping-treated plots. In contrast, Daewon showed reduced ABS/RC and TR_0_ under plant topping. In 2022, only PI_ABS_ differed among cultivars (*p* = 0.0091), with Taegwang recording the highest and Daewon the lowest values.

### 2.5. Yield Component and Grain Yield

Soybean yield components and grain yield were evaluated over two years (Table 8). The number of branches was not affected by the plant topping treatment or the cultivar in 2021; however, it showed a significant difference among cultivars in 2022. The node number was lower under the plant topping-treated than under the non-plant topping-treated in both years (*p* < 0.0001 for 2021; *p* = 0.0003 for 2022). Notably, in 2022, a significant interaction between plant topping treatment and cultivar was observed for node number (*p* = 0.0371). Daewon and Taegwang showed a greater node reduction under plant topping than other cultivars.

The 100-grain weight was higher in Daechan (31.7 g) and Daewon (31.9 g) than in the other cultivars (*p* = 0.0012) in 2021. The number of pods was unaffected in 2021 but showed a plant topping treatment × cultivar interaction in 2022 (*p* = 0.0174). The plant topping treatment of Daewon and Jinpung reduced the number of pods compared to non-plant topping plots (by 30.3 and 30.2%, respectively), while Daechan and Taegwang had no significant difference. The number of grains in 2022 was affected only by cultivar (*p* = 0.0005). Daewon (129.1 per plant) and Jinpung (121.2 per plant) had the highest values.

Grain yield was generally higher in 2021 than in 2022. In both years, significant differences in grain yield were observed among cultivars (*p* = 0.0008 for 2021; *p* < 0.0001 for 2022). In 2021, Taegwang had the lowest yield (2601 kg ha^−1^) among the cultivars. In 2022, the grain yields of Daewon (2550 kg ha^−1^) and Jinpung (2700 kg ha^−1^) were greater than those of Daechan (1661 kg ha^−1^) and Taegwang (1571 kg ha^−1^). Notably, a significant cultivar × plant topping treatment interaction was detected in 2021 (*p* = 0.0087). The grain yield of Jinpung in the plant topping-treated was 34% higher than in the non-plant topping-treated (3621 vs. 2701 kg ha^−1^, respectively). Jinpung showed the lowest yield in non-plant topping-treated plots, but the highest in plant topping-treated plots. Although the overall effect of plant topping treatment was not significant in two years, the yield tended to be higher in plant topping-treated plots, indicating a cultivar-dependent response.

## 3. Discussion

### 3.1. Plant Height and Stem Diameter

Our study investigated the effect of plant topping on the aboveground growth of various soybean cultivars. Based on field experiments conducted in 2021 and 2022, plant height was measured at three growth stages for different cultivars (Table 2). Over the two-year study period, plant topping treatment consistently reduced soybean plant height at all growth stages, and the reduced plant height after plant topping was maintained until the late growth stage. The sustained reduction in plant height suggests a modification in shoot growth patterns, likely due to the suppression of apical dominance. This observation aligns with previous findings indicating that the removal of the terminal bud alters the hormonal balance between auxin and cytokinins, thereby inhibiting the elongation of the main stem [25,34,35]. Consistent with this, terminal bud removal at both the V4 and R1 stages of soybean significantly reduced plant height and lodging, regardless of treatment timing [24].

A significant interaction was also observed between cultivars and plant topping at the R1 stage in both years, indicating that the response to plant topping was cultivar-dependent. Daewon had a higher plant height than other cultivars and exhibited excessive growth. However, after the plant topping treatment, plant height at the R1 stage showed no significant difference from that of other cultivars, except for Daewon in 2022. This suggests that plant topping may be effective in suppressing excessive vegetative growth in tall cultivars with high elongation potential. Additionally, it may contribute to the formation of a more stable canopy architecture in such cultivars. This morphological change may be attributed not only to the suppression of apical dominance via reduced auxin levels, but also to a shift in assimilate allocation. Specifically, resources may have been redirected toward reproductive organs, contributing to the development of a shorter and more compact canopy [23,26].

Although lodging was not directly assessed in our study, plant topping consistently reduced plant height across all developmental stages. Previous studies have reported that soybean plants become more susceptible to lodging when the main stem elongates between the V6 and R1 stages [36], or when internode elongation occurs beyond the fifth node [37]. It has also been reported that lodging severity increases as the proportion of non-pinched rows rises in an alternative row pinching system, highlighting the importance of managing vertical shoot growth to reduce lodging risk [14]. In our study, the timing and sustained effect of plant topping observed may offer potential benefits in mitigating lodging risk. Overall, plant topping effectively controlled plant height, with implications for improved shoot structure and potential lodging resistance, especially in tall cultivars, such as Daewon. However, further studies are needed to directly evaluate its impact on lodging under diverse field conditions.

Stem diameter before and after plant topping treatment was also measured over the two years (Table 3). In 2021, stem diameter showed no significant difference among treatments. However, at the R1 stage in 2022, except for Taegwang, the stem diameter of the plant topping-treated plot was lower than that of the non-plant topping-treated. However, at the R2 stage, there was no significant difference between plant topping treatments. This suggests that plant topping tends to reduce stem diameter at R1 stages, but no statistical difference was observed in the later season. Additionally, unlike others, little effect of plant topping on the stem diameter was observed for Taegwang, indicating the response to plant topping varies by cultivar. While there were substantial studies on the effects of plant topping for plant height of soybean, there has been no previous research on stem diameter.

### 3.2. LAI and NDVI

LAI was estimated at three growth stages over two years after the plant topping treatment (Table 4). Overall, LAI in 2022 tended to be slightly higher than in 2021. This trend appears to be associated with the shorter plant height observed in 2021, which was likely caused by excessive rainfall that year, subsequently affecting LAI. Except for the R3 stage in 2022, LAI in the plant topping-treated plots was lower than that in the non-plant topping-treated plots across all growth stages in both years, and the difference persisted into the later stages of growth.

Light interception by leaves varies significantly according to the LAI [38], and the photosynthetic rate of the leaf canopy in many crops is limited by light [39]. As LAI increases, the upper leaves create substantial shading over the lower leaves, thereby significantly reducing light penetration into the lower canopy [40,41]. In our study, the LAI of the non-plant topping-treated plots was higher than that of the plant topping-treated plots, potentially limiting photosynthetic capacity in the lower canopy due to greater light interception by the upper leaves [42,43]. However, plant topping treatment removed the upper leaf layer, thereby forming a canopy structure that allowed sufficient light penetration and enabled effective photosynthesis in the lower leaves. By altering the shoot architecture, plant topping reduced LAI and promoted favorable conditions for carbon assimilation in the lower part of the plant. Similar physiological responses were reported in cotton. Plant topping improved canopy carbon assimilation and promoted photoassimilate allocation to reproductive organs in cotton, increasing the yield and harvest index [23,26]. Modifying the canopy structure through plant topping can regulate source–sink balance under varying plant densities [44]. These are in line with the reduction in LAI and structural adjustment of the canopy induced by plant topping can improve light capture and resource use efficiency in the current study.

Since LAI influences canopy photosynthesis, an optimal LAI can result in higher grain yield [45]. LAI values within the range of 4.5–5.5 are positively correlated with soybean yield [46]. An optimal LAI range of 3.5 to 4.0 at the R1 stage and an optimum value of 4.5 were reported for maximizing soybean yield [47,48]. Consistent with these previous studies, the LAI of plant topping-treated soybeans in our study reached 4.5 at R2 in 2021 and 5.0 at R1 in 2022, both within the reported optimal range. Although excessive LAI can intensify shading in the lower canopy and extend vegetative growth [48], our findings suggest that plant topping may help mitigate these limitations by altering canopy structure. Plant topping may enhance light penetration and improve conditions for photosynthesis in the lower canopy. Nonetheless, further studies are needed to directly assess the photosynthetic response along with these structural changes.

Based on the results from 2021 and 2022, NDVI did not differ significantly between the plant topping-treated plots and non-treated plots at R2 (2021) and R1 (2022) (Table 5). At the later growth stages, NDVI was higher in the plant topping-treated plots than in the non-treated plots. NDVI values of soybean tend to decline in the later growth stages due to leaf senescence [49]. However, in our study, plant topping treatment delayed senescence, allowing green leaves to be maintained until the R3–R4 stages. These findings indicate that then plant topping treatment extended the photosynthetically active period of the soybean plant, while recovering LAI in the later season (Table 4).

### 3.3. Chlorophyll Concentration and Chlorophyll Fluorescence

In two years, chlorophyll concentration was estimated, revealing a temporary decline at the R1 stage in 2021 under the plant topping treatment, with no significant differences observed at later growth stages (Table 6). Decapitation in soybean was reported to induce a stay-green phenotype and delayed senescence by increasing chlorophyll content [27]. Similarly, 15% defoliation was reported to enhance leaf greenness and delay senescence, ultimately improving yield in soybean, whereas severe defoliation over 30% reduced leaf greenness [15]. Unlike these previous findings, our study showed that the chlorophyll concentration temporarily decreased at the R1 stage under plant topping in 2021 but subsequently recovered. Plant topping in cotton promoted compensatory growth by stimulating the formation of new branches [44], and enhanced cytokinin accumulation, resulting in altered physiological traits that facilitated branch development [50]. These findings support the idea that the temporary reduction in chlorophyll concentration observed in our study reflects a transient stress response induced by apical bud removal. The subsequent recovery may represent a physiological adjustment that compensates for apical damage.

Chlorophyll fluorescence parameters were measured at the R1 stage for 2 years (Table 7). In 2021, significant interactions between plant topping treatment and cultivar were observed for ABS/RC, TR_0_/RC, and DI_0_/CS. Notably, only Taegwang exhibited higher ABS/RC, TR_0_/RC, and DI_0_/CS values in the plant topping-treated plots than in the non-treated plots. Previous studies have reported that abiotic stress reduces the number of active PSII reaction centers, resulting in increased values of chlorophyll fluorescence parameters, such as ABS/RC, TR_0_/RC, ET_0_/RC, and DI_0_/RC, which indicate energy flux per PSII reaction center [33,51,52]. In our study, Taegwang showed increased ABS/RC and TR_0_/RC under plant topping treatment in 2021, unlike the other cultivars. This suggests that Taegwang remained in a stress condition longer than other cultivars following plant topping treatment, potentially resulting in structural or functional impairments in PSII. In addition, Taegwang exhibited a significantly lower plant height at the R3 stage in 2021 compared to the other cultivars (Table 2), suggesting a slower recovery in vegetative growth after plant topping. These findings imply that photochemical responses to plant topping treatment may vary among cultivars and could be associated with aboveground growth characteristics, such as plant height.

### 3.4. Yield Component and Grain Yield

The yield and yield components among cultivars under plant topping treatment were estimated (Table 8). In 2022, a significant interaction was observed between plant topping treatment and cultivar for the node number. The node number of Taegwang and Daewon decreased under plant topping treatment compared to the non-plant topping treatment. However, for two years, the number of branches was not significantly affected by the plant topping treatment.

Previous studies reported that plant topping treatment reduced the node number on the main stem while increasing the node number on the branches in legume crops [25,53] The plant topping treatment on soybean during the vegetative stage increased the number of branches or branch length [27,28,54]. This response has been attributed to the removal of the apical bud, which suppresses the main stem growth and activates dormant axillary buds, leading to the development of new branches. In contrast to previous studies, plant topping in our study did not increase branch number. Nonetheless, the observed reduction in main stem node number in Daewon and Taegwang suggests that apical dominance was suppressed. However, the expected compensatory increase in branch development was not evident. Plant topping was reported to enhance cytokinin accumulation in branches, thereby promoting branch growth and increasing branch number [27,50]. However, the physiological explanation for the branch number responses in our study remains limited.

The number of pods in Daewon and Jinpung exhibited a 30% reduction under the plant topping treatment in 2022; however, the number of grains showed no significant difference in response to the plant topping treatment. Grain yield was higher in 2021 than in 2022, likely due to reduced precipitation during the mid-growth stage of soybeans in July and August 2022 (Table 1). In 2021, grain yield showed significant differences among cultivars under the plant topping treatment. Jinpung, which had the lowest yield within the non-plant topping-treated plots, recorded the highest yield within the plant topping-treated plots, showing a 34% increase and indicating that its yield was higher in the plant topping-treated plots than in the non-treated plots. Other cultivars showed no significant yield response to the plant topping.

Previous studies have reported that plant topping at the vegetative or early reproductive stages can suppress apical dominance, promote lateral branch outgrowth, and ultimately increase soybean yield [24,27,28,34,54]. Although plant topping treatment did not increase the number of branches or yield in the current study, it reduced plant height (Table 2) and the node number on the main stem (Table 8), thereby contributing to the formation of a more structurally stable plant architecture. Other studies have simulated the defoliation caused by hail or pests during reproductive stages, such as seed filling, and reported corresponding yield reductions [55,56,57]. Yield responses varied with defoliation levels. No yield loss was observed at 67% defoliation at the R2 stage, but researchers observed a reduction at 100% defoliation [58]. However, in the present study, plant topping treatment did not result in soybean yield reduction. Unlike previous studies that reported yield loss due to excessive leaf removal reducing the source capacity for reproductive sinks [59], our treatment was conducted at the V6-7 stage by cutting the stem 30–35 cm above the ground, which left sufficient source to support seed filling. A 15% defoliation improved soybean yield by enhancing photosynthesis and leaf greenness, while defoliation above 30% reduced the yield due to insufficient recovery of leaf area and growth [15]. Similarly, our results suggest that a moderate level of plant topping enabled the recovery of plant height and LAI (Table 2 and Table 4), ensuring adequate light interception in the lower canopy and sufficient allocation of assimilates to pods and seeds, thus preventing yield loss. Plant topping in soybean induced stay-green traits, preserved chlorophyll content, and maintained photosynthetic capacity, which enhanced reproductive potential and yield [27]. In our study, chlorophyll concentration was temporarily reduced by plant topping but later recovered through compensatory growth (Table 6), suggesting that while the plant topping had a transient effect, it likely did not impair photosynthesis and, thus, did not contribute to yield reduction. Also, the effect of plant topping treatment on soybean yield varied depending on the cultivar in this study. Most cultivars showed no significant responses to plant topping treatment, but in 2021, the grain yield of Jinpung exhibited a substantial increase in plant topping treatment. As a result of plant topping, Jinpung showed reduced plant height (Table 2), contributing to a more balanced aboveground structure. Moreover, LAI remained relatively stable compared to other cultivars through the late developmental stages (Table 4), potentially enhancing light penetration to the lower canopy and improving the supply of photoassimilates to the seed. These findings suggest that Jinpung may be well-suited to plant topping. However, the physiological basis of these responses remains unclear, requiring further study to clarify cultivar-specific differences. Nevertheless, plant topping represents a practical and accessible technique for small-scale farmers, offering a potential means to maintain yield while improving lodging resistance.

## 4. Materials and Methods

### 4.1. Experimental Site and Soil Chemical Compositions

The current field experiment was conducted at Gyeongsang National University (GNU)’s off-campus Research Farm in Jinju, Korea (35°14′ N 128°09′ E 16 m a.s.l.). The soils were sandy loams (68% sand, 30% silt, and 2% clay-sized particles). Before and after the experiment of each year, soil samples were collected using a soil auger (30 cm in depth and 2.54 cm in diameter). Up to sixteen soil samples were collected in a “W” pattern and then composited. Soil chemical properties were analyzed during 2021–2022. The soil pH was 5.88 and the organic matter was 10.13 g kg^−1^ (Table 9). The weather data during two growing seasons (2021–2022) were obtained from the Korea Meteorological Administration (Available online: https://data.kma.go.kr/, (accessed on 24 September 2024), (in Korean)). The monthly mean temperature, growing degree days (GDD) with 10 °C base temp, rainfall for the field study period, total amount of solar radiation (MJ m^−2^), and duration of sunshine (h) were recorded.

For the field preparation, the entire field was plowed then rotovated. For a better aeration during the monsoon season, a 25 cm ridges 90 cm apart were prepared with a small-size multipurpose cultivator (MR1200, ASIA Technology, Daegu, Republic of Korea). The experiment was conducted using a randomized complete block design (RCBD) with four replications.

The plot size in 2021 was 8.5 m × 4 m, and the plot size in 2022 was 7.5 m × 4 m. For seed stain (*Cercospora kikuchii*) control, soybean seeds were treated with a fungicide, Saechong (Hankook Samgong, Seoul, Republic of Korea; thiram 26.5%), at a rate of 30 mL per kg ^−1^ of seed. Soybean seeds were planted at a rate of two seeds per hill, on the 15 cm ridge, with intra-row spacing using a manual disk planter (TP–10RA, Korea Agritechno Search Corp., Cheongju, Republic of Korea), with a target density of 150,000 seeds ha^−1^. The herbicide alachlor (2.0 kg a.i. ha^−1^) was applied immediately following planting for early season weed management. Other management practices followed the guidelines of agricultural standard cultural practices of the Korea Rural Development Administration (RDA, 2003) [60].

The cultivars used for the experiment in this study were Daechan, Daewon, Jinpung, and Taegwang. Among them, Daechan has a stem length that is 11.7% shorter than Daewon and a pod-setting height that is 25% higher, which contributes to its resistance to lodging and makes it well-suited for mechanical harvesting. Daewon exhibits longer stem length and a higher number of branches, and it is the most widely grown cultivar in the nation. Jinpung exhibits a 9.0% shorter stem length and a 25% higher pod-setting height compared to Daewon, providing superior lodging resistance and suitability for mechanical harvesting. Taegwang is characterized by superior yield and a higher 100-grain weight. The cultivars included in the study have similar growth periods and exhibit high cultivation stability, enabling consistent growth across various conditions. Furthermore, these cultivars are among the major varieties grown in Korea [61].

Plant topping was conducted using a pole hedge trimmer (DUN500W, Makita, Japan) at the V7 stage in 2021 (21 July) and the V6 stage in 2022 (12 July), targeting a cutting height within range of 30 to 35 cm above the soil surface to remove the bud of the apical meristem (RDA soybean handbook 2021 in Korean) [62].

### 4.2. Measurements of Crop Traits

The aboveground growth of soybean plants was assessed by measuring plant height and stem diameter every two weeks, starting four weeks after planting. Plant height was measured using a stick ruler from the ground surface to the uppermost leaf. Stem diameter was measured at the base of the stem using a digital vernier caliper. For each parameter, 10 plants per plot were randomly selected from the middle two rows.

Leaf area index (LAI) data were collected approximately every two weeks from the V7 to R6 stages, starting two weeks after plant topping. Soybean LAI measurements were obtained from the two center rows to minimize the border effect. The LAI of each plot was recorded using a plant canopy analyzer (LAI2200-C, Li-Cor Biosciences, Lincoln, NE, USA) with a 90° view angle cap and six under-canopy readings. Measurements were taken at equal distance perpendicular to the rows in a “W” pattern. NDVI was measured using an active crop canopy sensor (Crop Circle ACS-430, Holland Scientific, Lincoln, NE, USA). The ACS-430 measures at wavelengths of 670 nm, 730 nm, and 780 nm, and the output is 10 measurements per second. The ACS-435 has a field of view of 40° by 10°. Chlorophyll concentration was measured on fully developed uppermost leaves of 10 plants per plot using a chlorophyll content meter (CCM–300, Opti-Sciences, Hudson, NH, USA), which records chlorophyll concentration on a surface basis (mg·m^−2^). The photochemical efficiency of photosystem II was estimated by a portable fluorometer (OS30p^+^, Opti-Science, Hudson, NH, USA). Clips were applied on two locations of the upper fully-grown leaves. After 20 min of dark adaptation, minimum (F_0_), maximum (F_m_), and variable (F_v_) fluorescence emissions were measured on leaves. The efficiency of PSII was expressed as the F_v_/F_m_ ratio. Additionally, the OJIP fluorescence transient curve was obtained. From the OJIP fluorescence curve, key fluorescence parameters were derived, including the minimum fluorescence (O-step), fluorescence at 0.3 ms (K-step), 2 ms (J-step), 30 ms (I-step), and the maximum fluorescence (P-step). These measurements were used to calculate chlorophyll fluorescence parameters, such as ABS/RC, TR_0_/RC, ET_0_/RC, and DI_0_/CS [63]. A detailed list of formulas and parameter definitions employed in the JIP test is provided in Table S1 [64].

### 4.3. Yield Components and Yield

As each cultivar had a different rate of maturity, sampling dates for yield components differed, and were the 6 October (Taegwang), 13 October (Daechan), and 19 October, (Daewon, Jinpung) in 2021, and the 21 October (Daechan), and 28 October (Daewon, Jinpung, Taegwang) in 2022, respectively. Ten representative plants from each plot were selected, then the node number per plant, the number of pods, the number of branches per plant, the number of grains, and 100-grain weight were recorded. A total area of 4.5 m^2^ was harvested from the middle rows for grain yield analysis. After manual threshing and cleaning, the processed seeds were used to estimate the final grain yield (adjusted to a 14% moisture content).

### 4.4. Statistical Analysis

The analysis was conducted using the PROC MIXED model in SAS v9.4 software (SAS Institute Inc., Cary, NC, USA), with plant topping treatment and cultivar set as fixed effects, and block as a random effect. A two-way analysis of variance (ANOVA) was performed to evaluate significance, and significance testing was conducted at the 5% level.

## 5. Conclusions

The current study evaluated the effects of plant topping on growth characteristics, physiological responses, and yield of four major soybean cultivars in Korea for two years. Plant topping consistently reduced plant height and LAI through the later growth stages. This reduction may improve light penetration into the lower canopy. Cultivar-specific differences were also observed in photochemical responses. In particular, Taegwang showed increased ABS/RC, TR_0_/RC, and DI_0_/CS values under plant topping, indicating greater sensitivity to plant topping-induced stress. Although grain yield was not significantly affected in other cultivars, Jinpung showed a 34% increase in grain yield following plant topping in 2021 only, when rainfall was relatively higher than in 2022. While testing in one location is a limitation of the current study, these findings demonstrate that plant topping can alter plant architecture in a way that potentially mitigates lodging risk and enhances photosynthetic efficiency in the lower canopy. Plant topping may serve as a practical and accessible management option for smallholder farmers aiming to manage risk and maintain yield stability under unfavorable weather conditions, such as mid-season rainfall events. Future research will include multilocational trials across diverse environments. Direct assessments of lodging risk, analysis of root architecture, and hormonal regulations are also needed.

## Figures and Tables

**Table 1:** Monthly mean temperature, total rainfall, growing degree days (GDDs), total amount of solar radiation, and duration of sunshine in Jinju from 2021 to 2022.

		Jun.	Jul.	Aug.	Sep.	Oct.	Total
Mean temperature (°C)	2021	22.2	26.2	26.0	21.9	15.9	-
	2022	22.7	26.5	26.3	21.8	15.1	-
Total rainfall (mm)	2021	101	359	320	122	46	948
	2022	121	218	157	167	22	685
GDDs (°C) *	2021	376	516	517	369	173	1951
	2022	394	526	515	365	96	1896
Total amount of solar radiation (MJ m^−2^)	2021	586	590	525	370	449	2520
	2022	574	596	481	443	433	2527
Duration of sunshine (h)	2021	207	228	196	140	238	1009
	2022	206	224	174	176	231	1011

* Base temperature for GDDs was 10 °C.

**Table 2:** Effects of soybean cultivar × plant topping management on plant height at different developmental stages in 2021 and 2022.

Plant Height (cm)								
		V6	R1			R3		
Year	Cultivar	Before topping	Non-topping	Topping	Mean	Non-topping	Topping	Mean
2021	Daechan	35.1 C ^†^	54.8 Ba	45.9 Ab	50.3 B	72.6 ^§^	63.1	67.9 BC
	Daewon	43.2 A	66.1 Aa	49.4 Ab	57.7 A	82.4	67.3	74.8 A
	Jinpung	38.4 B	56.1 Ba	46.0 Ab	51.0 B	74.9	66.7	70.8 AB
	Taegwang	38.8 B	57.1 Ba	46.1 Ab	51.6 B	70.0	54.6	62.3 C
	Mean		58.5 a ^‡^	46.8 b		75.0 a	62.9 b	
		**V6**	**R1**			**R2**		
Year	Cultivar	Before topping	Non-topping	Topping	Mean	Non-topping	Topping	Mean
2022	Daechan	30.0 C	52.5 Ca	40.5 Bb	46.5 C	75.1	62.6	68.9 C
	Daewon	41.1 A	78.1 Aa	53.8 Ab	65.9 A	99.6	83.0	91.3 A
	Jinpung	34.4 B	60.4 Ba	52.0 Ab	56.2 B	80.3	73.4	76.9 B
	Taegwang	36.7 B	73.2 Aa	55.7 Ab	64.4 A	95.7	80.6	88.2 A
	Mean		66.0 a	50.5 b		87.7 a	74.9 b	

^†^ Within a column, values followed by different uppercase letters are significantly different among cultivars at *p* ≤ 0.05. ^‡^ Within a row, values followed by different lowercase letters are significantly different among plant topping treatments at *p* ≤ 0.05. ^§^ Statistically not significant (*p* > 0.05).

**Table 3:** Effects of soybean cultivar × plant topping management on stem diameter at different developmental stages in 2021 and 2022.

Stem Diameter (mm)								
		V6	R1			R3		
Year	Cultivar	Before topping	Non-topping	Topping	Mean	Non-topping	Topping	Mean
2021	Daechan	7.2 ^†^	10.3	9.6	10.0	12.5	12.4	12.4
	Daewon	6.7	9.1	8.8	9.0	12.0	11.1	11.5
	Jinpung	7.0	9.9	9.0	9.5	11.9	11.3	11.6
	Taegwang	7.3	9.5	9.4	9.4	11.4	11.7	11.5
	Mean		9.7	9.2		11.9	11.6	
		**V6**	**R1**			**R2**		
Year	Cultivar	Before topping	Non-topping	Topping	Mean	Non-topping	Topping	Mean
2022	Daechan	5.9 C ^‡^	8.8 Ba	7.3 Cb	8.0 C	10.9	10.6	10.8 C
	Daewon	6.9 A	9.9 Aa	8.7 ABb	9.3 A	12.4	11.7	12.1 AB
	Jinpung	6.1 BC	9.3 ABa	8.2 Bb	8.8 B	11.7	11.4	11.6 B
	Taegwang	6.6 AB	9.1 Ba	9.5 Aa	9.3 A	12.6	12.5	12.5 A
	Mean		9.3 a ^§^	8.4 b		11.9	11.6	

^†^ Statistically not significant (*p* > 0.05). ^‡^ Within a column, values followed by different uppercase letters are significantly different among cultivars at *p* ≤ 0.05. ^§^ Within a row, values followed by different lowercase letters are significantly different among plant topping treatments at *p* ≤ 0.05.

**Table 4:** Effects of soybean cultivar × plant topping management on leaf area index (LAI) at different developmental stages in 2021 and 2022.

		R2			R4			R6		
Year	Cultivar	Non-topping	Topping	Mean	Non-topping	Topping	Mean	Non-topping	Topping	Mean
2021	Daechan	5.4 ^†^	4.7	5.0	6.6	5.8	6.2 A ^§^	6.1	5.2	5.7 A
	Daewon	4.9	4.1	4.5	6.2	5.2	5.7 B	5.9	4.9	5.4 A
	Jinpung	5.4	4.8	5.1	6.6	5.9	6.3 A	6.1	5.4	5.7 A
	Taegwang	5.3	4.4	4.8	6.0	5.2	5.6 B	5.1	4.4	4.8 B
	Mean	5.3 a ^‡^	4.5 b		6.4 a	5.5 b		5.8 a	5.0 b	
		**R1**			**R3**			**R5**		
Year	Cultivar	Non-topping	Topping	Mean	Non-topping	Topping	Mean	Non-topping	Topping	Mean
2022	Daechan	4.9	4.7	4.8 B	5.6	5.1	5.4 C	5.6	4.4	5.0 B
	Daewon	5.1	3.9	4.5 B	6.0	5.5	5.7 BC	6.1	4.9	5.5 B
	Jinpung	5.9	5.3	5.6 A	6.8	5.7	6.2 AB	6.4	6.1	6.3 A
	Taegwang	6.6	5.9	6.2 A	6.7	7.0	6.8 A	6.2	5.0	5.6 B
	Mean	5.6 a	5.0 b		6.3	5.8		6.1 a	5.1 b	

^†^ Statistically not significant (*p* > 0.05). ^‡^ Within a row, values followed by different lowercase letters are significantly different among plant topping treatments at *p* ≤ 0.05. ^§^ Within a column, values followed by different uppercase letters are significantly different among cultivars at *p* ≤ 0.05.

**Table 5:** Effects of soybean cultivar × plant topping management on NDVI at different developmental stages in 2021 and 2022.

		V7	R2			R4		
Year	Cultivar	Before topping	Non-topping	Topping	Mean	Non-topping	Topping	Mean
2021	Daechan	0.798 ^†^	0.863	0.865	0.864 BC ^‡^	0.871	0.876	0.873 D
	Daewon	0.845	0.896	0.892	0.894 A	0.894	0.897	0.896 B
	Jinpung	0.827	0.887	0.883	0.885 AB	0.901	0.902	0.901 A
	Taegwang	0.850	0.869	0.845	0.857C	0.886	0.895	0.891 C
	Mean		0.879	0.871		0.888 b ^§^	0.893 a	
		**V6**	**R1**			**R3**		
Year	Cultivar	Before topping	Non-topping	Topping	Mean	Non-topping	Topping	Mean
2022	Daechan	0.840	0.868	0.871	0.869 B	0.895	0.890	0.892 C
	Daewon	0.842	0.899	0.886	0.893 A	0.916	0.919	0.917 B
	Jinpung	0.727	0.902	0.901	0.902 A	0.925	0.927	0.926 A
	Taegwang	0.647	0.898	0.898	0.898 A	0.919	0.920	0.920 B
	Mean		0.892	0.889		0.914	0.914	

^†^ Statistically not significant (*p* > 0.05). ^‡^ Within a column, values followed by different uppercase letters are significantly different among cultivars at *p* ≤ 0.05. ^§^ Within a row, values followed by different lowercase letters are significantly different among plant topping treatments at *p* ≤ 0.05.

**Table 6:** Effects of soybean cultivar × plant topping management on chlorophyll concentration at different developmental stages in 2021 and 2022.

Chlorophyll Concentration (mg m^−2^)										
		R1			R3			R6		
Year	Cultivar	Non-topping	Topping	Mean	Non-topping	Topping	Mean	Non-topping	Topping	Mean
2021	Daechan	286.8 ^†^	256.5	271.6 B ^§^	272.5	286.8	279.6 C	377.0	369.5	373.3
	Daewon	307.8	278.8	293.3 A	348.0	368.5	358.3 A	425.5	399.3	412.4
	Jinpung	288.3	275.0	281.6 AB	268.0	283.5	275.8 C	450.5	423.5	437.0
	Taegwang	308.3	280.8	294.5 A	309.8	297.5	303.6 B	397.0	382.8	389.9
	Mean	297.8 a ^‡^	272.8 b		299.6	309.1		412.5	393.8	
		**R1**			**R2**			**R4**		
Year	Cultivar	Non-topping	Topping	Mean	Non-topping	Topping	Mean	Non-topping	Topping	Mean
2022	Daechan	335.8	342.8	339.3	327.3	336.2	324.6	311.8	328.5	320.1
	Daewon	362.3	341.3	351.8	337.3	338.0	337.6	331.0	339.0	335.0
	Jinpung	352.3	345.3	348.8	334.8	326.3	330.5	314.8	307.8	311.3
	Taegwang	348.8	349.0	348.9	345.5	335.8	340.6	333.3	335.8	334.5
	Mean	349.8	344.6		336.2	330.5		322.7	327.8	

^†^ Statistically not significant (*p* > 0.05). ^‡^ Within a row, values followed by different lowercase letters are significantly different among plant topping treatments at *p* ≤ 0.05. ^§^ Within a column, values followed by different uppercase letters are significantly different among cultivars at *p* ≤ 0.05.

**Table 7:** Effects of soybean cultivar × plant topping management on chlorophyll fluorescence parameters at R1 in 2021 and 2022.

	F_v_/F_m_				PI_ABS_			ABS/RC		
Year	Cultivar	Non-topping	Topping	Mean	Non-topping	Topping	Mean	Non-topping	Topping	Mean
2021	Daechan	0.673 ^†^	0.692	0.683	1.909	2.504	2.207	2.048 A ^‡^ a ^§^	1.941 Ba	1.995
	Daewon	0.667	0.657	0.662	1.728	2.153	1.940	2.186 Aa	1.776 Bb	1.981
	Jinpung	0.656	0.690	0.673	1.611	1.935	1.773	1.914 Aa	1.985 Ba	1.950
	Taegwang	0.709	0.667	0.688	2.569	1.805	2.187	1.919 Ab	2.329 Aa	2.124
	Mean	0.676	0.676		1.954	2.099		2.017	2.008	
	**TR** **_0_/RC**				**DI** **_0_/CS**			**ET** **_0_/RC**		
	Cultivar	Non-topping	Topping	Mean	Non-topping	Topping	Mean	Non-topping	Topping	Mean
	Daechan	0.314 ABa	0.296 Ba	0.305	0.671 Aa	0.598 Ba	0.635	0.891	0.899	0.895
	Daewon	0.338 Aa	0.275 Bb	0.307	0.736 Aa	0.578 Ba	0.657	0.902	0.741	0.822
	Jinpung	0.295 Ba	0.309 ABa	0.302	0.659 Aa	0.647 ABa	0.653	0.765	0.828	0.797
	Taegwang	0.293 Bb	0.339 Aa	0.316	0.589 Ab	0.828 Aa	0.708	0.890	0.959	0.924
	Mean	0.310	0.305		0.664	0.663		0.862	0.857	
2022	**F_v_/F_m_**				**PI_ABS_**			**ABS/RC**		
	Cultivar	Non-topping	Topping	Mean	Non-topping	Topping	Mean	Non-topping	Topping	Mean
	Daechan	0.790	0.782	0.786	8.307	7.024	7.665 B	1.550	1.633	1.592
	Daewon	0.787	0.780	0.784	7.733	7.380	7.556 B	1.672	1.601	1.637
	Jinpung	0.789	0.797	0.793	8.947	8.434	8.691 AB	1.483	1.639	1.561
	Taegwang	0.789	0.794	0.792	8.986	10.251	9.619 A	1.527	1.392	1.459
	Mean	0.789	0.788		8.493	8.272		1.558	1.566	
	**TR** **_0_/RC**				**DI** **_0_/CS**			**ET** **_0_/RC**		
	Cultivar	Non-topping	Topping	Mean	Non-topping	Topping	Mean	Non-topping	Topping	Mean
	Daechan	0.218	0.239	0.229	0.325	0.358	0.341	0.940	0.954	0.947
	Daewon	0.238	0.233	0.236	0.357	0.354	0.355	0.999	0.933	0.966
	Jinpung	0.208	0.237	0.222	0.314	0.335	0.324	0.900	0.993	0.946
	Taegwang	0.212	0.194	0.203	0.320	0.288	0.304	0.931	0.851	0.891
	Mean	0.219	0.226		0.329	0.333		0.943	0.933	

^†^ Statistically not significant (*p* > 0.05). ^‡^ Within a column, values followed by different uppercase letters are significantly different among cultivars at *p* ≤ 0.05. ^§^ Within a row, values followed by different lowercase letters are significantly different among plant topping treatments at *p* ≤ 0.05.

**Table 8:** Effects of soybean cultivar × plant topping management on yield components and grain yield in 2021 and 2022.

		Branch No. (Plant^−1^)			Node No. (Plant^−1^)			100-Grains Weight (g)		
Year	Cultivar	Non-topping	Topping	Mean	Non-topping	Topping	Mean	Non-topping	Topping	Mean
2021	Daechan	0.55 ^†^	1.29	0.92	14.5	10.4	12.4 AB ^§^	32.1	31.3	31.7 A
	Daewon	0.65	1.20	0.93	13.7	10.6	12.2 B	33.8	30.0	31.9 A
	Jinpung	1.95	0.85	1.40	14.7	13.2	13.9 A	28.5	28.2	28.4 B
	Taegwang	1.45	1.55	1.50	13.5	9.3	11.4 B	28.8	28.8	28.8 B
	Mean	1.15	1.22		14.1 a ^‡^	10.9 b		30.8	29.6	
		**Pod no. (plant^−1^)**			**Grain no. (plant^−1^)**			**Grain yield (kg ha^−1^)**		
	Cultivar	Non-topping	Topping	Mean	Non-topping	Topping	Mean	Non-topping	Topping	Mean
	Daechan	81.6	80.8	81.2	133.0	140.2	136.6	3380 Aa	3520 Aa	3450 A
	Daewon	96.0	81.9	88.9	152.8	127.9	140.3	3661 Aa	3200 Aa	3430 A
	Jinpung	99.5	77.4	88.4	163.7	132.2	148.0	2701 Bb	3621 Aa	3161 A
	Taegwang	75.7	76.3	76.0	114.3	118.4	116.3	2731 Ba	2470 Ba	2601 B
	Mean	88.2	79.1		140.9	129.7		3120	3201	
2022		**Branch no. (plant^−1^)**			**Node no. (plant^−1^)**			**100-grains weight (g)**		
	Cultivar	Non-topping	Topping	Mean	Non-topping	Topping	Mean	Non-topping	Topping	Mean
	Daechan	0.85	0.55	0.70 B	13.9 ABa	13.5 Aa	13.7 A	29.4	28.0	28.7
	Daewon	1.45	2.15	1.80 A	13.9 ABa	10.2 Bb	12.0 B	31.6	30.3	31.0
	Jinpung	2.05	1.58	1.81 A	15.1 Aa	14.4 Aa	14.7 A	25.9	26.0	25.9
	Taegwang	1.99	1.86	1.93 A	12.6 Ba	10.0 Bb	11.3 B	29.6	31.1	30.4
	Mean	1.58	1.53		13.9 a	12.0 b		29.1	28.9	
		**Pod no. (plant^−1^)**			**Grain no. (plant^−1^)**			**Grain yield (kg ha^−1^)**		
	Cultivar	Non-topping	Topping	Mean	Non-topping	Topping	Mean	Non-topping	Topping	Mean
	Daechan	67.1 Ba	67.7 Aa	67.4 BC	95.5	97.7	96.6 B	1590	1741	1661 B
	Daewon	109.5 Aa	76.3 Ab	92.9 A	139.4	118.8	129.1 A	2431	2670	2550 A
	Jinpung	91.1 Aa	63.6 Ab	77.4 B	130.1	112.2	121.2 A	2620	2780	2700 A
	Taegwang	55.3 Ba	61.8 Aa	58.5 C	64.3	84.3	74.3 B	1500	1650	1571 B
	Mean	80.7a	67.4 b		107.3	103.2		2031	2210	

^†^ Statistically not significant (*p* > 0.05). ^‡^ Within a row, values followed by different lowercase letters are significantly different among plant topping treatments at *p* ≤ 0.05. ^§^ Within a column, values followed by different uppercase letters are significantly different among cultivars at *p* ≤ 0.05.

**Table 9:** Average soil chemical properties of the experimental site.

pH	EC	OM	Available P_2_O_5_	K	Ca	Mg
(1:5) ^†^	(dS m^−1^)	(g kg^−1^)	(mg kg^−1^)	Exchangeable (mg kg^−1^)		
5.88	0.13	10.13	88.13	0.17	2.64	0.49

^†^ Soil sample to water ratio of 1:5.

## Data Availability

The data presented in this study are available on request from the corresponding author due to the funding agency approval.

## Supplementary Materials

The following supporting information can be downloaded at: https://www.mdpi.com/article/10.3390/plants14132068/s1, Table S1: Summary of chlorophyll fluorescence parameters used by the JIP test for the analysis of the fluorescence transient O-J-I-P. Ref. [64] is cited in the supplementary materials.
