# Supplementary material for: Effect of Plant Topping on Seasonal Development, Physiological Changes, and Grain Yield of Soybean
Source: Plants (Basel). 2025 Jul 6;14(13):2068. doi: 10.3390/plants14132068 (PMC12251880; doi:10.3390/plants14132068)
Supplement: Supplementary file 1 [file plants-14-02068-s001.zip › plants-3713393-supplementary.pdf]

Supplementary Table S1 Summary of chlorophyll fluorescence parameters used by the JIP test for the analysis of the fluorescence transient O-J-I-P

**Derived Fluorescence Parameters**	**Meaning**
$F_0 \cong F_{50\mu s}$ or $\cong F_{20\mu s}$	Minimal fluorescence, when all PS II reaction centers are open
$F_M = F_P$	Maximal fluorescence, when all PS II reaction centers are closed
$M_0 \equiv (\Delta V/\Delta t)_0 \equiv 4\ (F_{300\mu s} - F_0)/(F_M - F_0)$	Approximated initial slope (in ms $^{-1}$) of the fluorescence transient
$V_J = (F_J - F_0)/(F_M - F_0)$	Relative variable fluorescence at the J-step
**Flux ratios**	
$\varphi_{Po} = F_V/F_M = [1 - (F_0/F_M)]$	Maximum quantum yield of primary photochemistry
$\psi_0 \equiv ET_0/TR_0 = (1 - V_J)$	Probability that a trapped exciton moves an electron into the electron transport chain beyond Q_A
**Specific energy fluxes**	
$ABS/RC = M_0\ (1/V_J)\ (1/\varphi_{Po})$	Absorption flux per reaction center
$TR_0/RC = M_0\ (1/V_J)$	Trapped energy flux per reaction center
$ET_0/RC = M_0\ (1/V_J)\ \psi_0$	Electron transport flux per reaction center
**Phenomenological energy fluxes**	
$TR_0/CS = \varphi_{Po}\ (ABS/CS)$	Trapped energy flux per cross section
$ET_0/CS = \varphi_{Eo}\ (ABS/CS)$	Electron transport flux per cross section
$DI_0/CS = (ABS/CS) - (TR_0/CS)$	Dissipated energy flux per cross section
**Performance index**	
$PI_{ABS} \equiv (RC/ABS)\ \{\varphi_{Po}/(1-\varphi_{Po})\}\ \{\psi_0/(1-\psi_0)\}$	Performance index on absorption basis
